# Supplementary material for: Bioactive Terpenes from Marine Sponges and Their Associated Organisms
Source: Mar Drugs. 2025 Feb 21;23(3):96. doi: 10.3390/md23030096 (PMC11943499; doi:10.3390/md23030096)
Supplement: Supplementary file 1 [file marinedrugs-23-00096-s001.zip › marinedrugs-3461541-supplementary.pdf]

Supplementary Materials

Bioactive Terpenes from Marine Sponges and their associated organisms

Yuan Yuan [1†], Yu Lei [1†], Muwu Xu [2], Bingxin Zhao [1,*] and Shihai Xu [1,*]

[1] Department of Chemistry, College of Chemistry and Materials Science, Jinan University, Guangzhou 510632, China; yuany@stu2024.jnu.edu.cn; 31000652@scau.edu.cn

[2] Department of Epidemiology and Environmental Health, School of Public Health and Health Professions, University at Buffalo, Buffalo, NY, USA. muwuxu@buffalo.edu

[*] Correspondence: bxzhao@jnu.edu.cn (B.Z.); txush@jnu.edu.cn (S.X.)

[†] These authors contributed equally to this work.

Table S1. Sesquiterpenes isolated from the marine sponges.

Compound	Sponge Species	Sponge-Derived Microbe	Sampling Location	Biological Activity	Ref.
3*β*,9*α*,11-trihydroxy-6-oxo-drim-7-ene (**1**)<br>2*α*,9*α*,11-trihydroxy-6-oxo-drim-7-ene (**2**)<br>2*α*,11-dihydroxy-6-oxodrim-7-ene (**3**)<br>(6-strobilactone-B) ester of (*E*, *E*)-6,7-dihydroxy-2,4-octadienoic acid (**6/7**)	*Suberites domuncula*	Fungus *Aspergillus ustus*	Adriatic Sea	No data	Liu et al., 2009 [20]
mono(6-strobilactone-B) ester of (*E*, *E*)-2,4-hexadienedioic acid (**4**)<br>(6-strobilactone-B) ester of (*E*, *E*)-6-oxo-2,4-hexadienoic acid (**5**)	*Suberites domuncula*	Fungus *Aspergillus ustus*	Adriatic Sea	Cytotoxic against L5178Y cells	-
(*E*)-6-(4′-hydroxy-2′-butenoyl)-strobilactone A (8)	*Psammocinia* sp.	Fungus *Aspergillus insuetus*	Israel	Cytotoxicity towards MOLT-4 human leukemia cells	Cohen et al., 2011 [21]
20-Epi-hydroxyhaterumadienone (**9**)<br>15-*Oxo*-puupehenoic acid (**10**)	*Hyrtios* sp.	No applicable	Papua New Guinea	No data	Robinson et al., 2009 [22]
(-)-Nakijinol E (**11**)<br>(–)-Dactylospongenones E (**12**)<br>5-epidactylospongenones E-F (**13–14**)	*Smenospongia aurea*、*Smenospongia cerebriformis, Verongula rigida*	No applicable	Key largo, Florida	No data	Hwang et al., 2015 [23]
(+)-5-*epi*-Nakijinol E (**15**)<br>Nakijinone A (**16**)<br>5-*epi*-Nakijinone A (**17**)<br>(+)-5-*epi*-20-*O*-Ethylsmenoquinone (**18**)	*Smenospongia aurea*、*Smenospongia cerebriformis, Verongula rigida*	No applicable	Key largo, Florida	Cytotoxic, antiproliferative	-
Aureol B (**19**)<br>Melemeleones C–D (**20–21**)<br>Cycloaurenones A–C (**22–24**)	*Dysidea* sp.	No applicable	Federated States, Micronesia	Inhibition against Na^+/K^+-ATPase, cytotoxic	Kim et al., 2015 [24]
Chartarolides A–C (**25–27**)	*Niphates recondite*	Fungus *Stachybotrys chartarum* WGC-25C-6	No data	Antitumor	Liu et al., 2017 [25]
Dactylospongenones G–H (**28–29**)	*Dactylospongia elegans*	No applicable	Indonesia	No data	Ebada et al., 2017 [26]
Pseudoceranoids A–J **(30–39)**	*Pseudoceratina purpurea*	No applicable	South China Sea	Cytotoxicity for **30–31**	Yu et al., 2023 [27]
5-*epi*-Nakijiquinone S (40)<br>5-*epi*-Nakijiquinone Q (**41**)<br>5-*epi*-Nakijiquinone T (**42**)<br>5-*epi*-Nakijiquinone U (**43**)<br>5-*epi*-Nakijiquinone N (**44**)	*Dactylospongia metachromia*	No applicable	Ambon, Indonesia	Cytotoxicity	Daletos et al., 2014 [28]

5-*epi*-Nakijinol C (**45**)<br>5-*epi*-Nakijinol D (**46**)	*Dactylospongia metachromia*	No applicable	Ambon, Indonesia	Cytotoxicity	-
5,8- *diepi*-Ilimaquinone (**47**)<br>4,5-*diepi*-Dactylospongiaquinone (**48**)	*Dactylospongia elegans*	No applicable	Palau and Malaysia	Activate HIF-1	Du et al., 2013 [29]
8-*epi*-Dactyloquinone B (**49**)<br>10,17-O-cyclo-4,5-diepi-Dactylospongiaquinone (**50**)<br>Cyclospongiacatechol (**51**)	*Dactylospongia elegans*	No applicable	Palau and Malaysia	No data	-
Hyrtiolacton A (**52**)<br>Nakijinol F (**53**)	*Hyrtios* sp.	No applicable	South China Sea	No data	Wang et al., 2017 [30]
Nakijinol G (**54**)	*Hyrtios* sp.	No applicable	South China Sea	PTP1B inhibition	-
Cinerols A–C, F (**55–57**, **60**)	*Dysidea cinerea*	No applicable	South China Sea	PTP1B, ATP-citrate lyase, and phosphatase-1 inhibition	Jiao et al., 2019 [31]
Cinerols D–E, G–K (**58–59**, **61–65**)	*Dysidea cinerea*	No applicable	South China Sea	No data	-
Aignopsanoic acid A (**66**)<br>Methyl aignopsanoate A (**67**)	*Cacospongia mycofijiensis*	No applicable	Northern Papua New Guinea	Active against *Trypanosoma brucei*	Johnson et al., 2009 [32]
Isoaignopsanoic acid A (**68**)	*Cacospongia mycofijiensis*	No applicable	Northern Papua New Guinea	No data	-
7α,8α-epoxy theonellin isothiocyanate (**69**)	*Phycopsis* sp.	No applicable	Mandapam coast, India	No data	Kondempudi et al., 2009 [33]
3-formamido-7,8-epoxy-α-bisabolane (**70**)<br>3-isocyano-7,8-epoxy-α-bisabolane (**71**)	*Axinyssa* sp.	No applicable	Hainan, China	No data	Sun et al., 2010 [34]
Aspergiterpenoid A (**72**)<br>(–)-Sydonol (**73**)<br>(–)-Sydonol acid (**74**)<br>(-)-5-(hydroxymethyl)-2-(2',6',6'-trimethyltetrahydro-2*H*-pyran-2-yl) phenol (**75**)	*Xestospongia testudinaria*	Fungus *Aspergillus* sp.	South China Sea	Antibacterial and *in vitro* cytotoxic	Li et al., 2012 [35]
Disydonols A–C (**76–78**)	*Xestospongia testudinaria*	Fungus *Aspergillus* sp.	South China Sea	Antibacterial and *in vitro* cytotoxic	Sun et al., 2012 [36]
Axinysaline A (**79**)	*Axinyssa* sp.	No applicable	Taiwan, China	Cytotoxicity against Molt 4 and K562	Liu et al., 2014 [37]
Axinysaline B (**80**)	*Axinyssa* sp.	No applicable	Taiwan, China	No data	-
7,10,11-Trihydroxy-8-en-3-formamidotheonellin (**81**)<br>7,11-Dihydroxy-10-methoxy-8-en-3-formamidotheonellin (**82**)	*Axinyssa variabilis*	No applicable	Hainan, China	No data	Liu et al., 2016 [38]

Halichonic acid B (**83**)	*Axinyssa* sp.	No applicable	Bajotalawaan, Sulawesi	No data	Hitora et al., 2021 [39]
Plakordiols A–D (**84–87**)<br>(7*R*, 10*R*)-hydroxycurcudiol (**88**)<br>(7*R*, 10*S*)-hydroxycurcudiol (**89**)	*Plakortis simplex*	No applicable	South China Sea	No data	Wang et al., 2021 [40]
Dysideamine (**90**)	*Dysidea* sp.	No applicable	Indonesia	Neuroprotective effect	Suna et al., 2009 [42]
21-Dehydroxybolinaquinone (**91**)	*Dysidea villosa*	No applicable	Hainan, China	PTP1B inhibition and cytotoxicity	Li et al., 2009 [43]
Nakijiquinones E–F (**92–93**)	*Spongia* sp.	No applicable	Okinawan, Japan	No data	Takahashi et al., 2009 [44]
Nakijiquinones J–R (**94–102**)	*Spongiidae* sp. SS-1047, SS-265, SS-1208	No applicable	Okinawan, Japan	No data	Takahashi et al., 2010 [45]
Dysidavarone A (**103**)<br>Dysidavarone D (**104**)	*Dysidea avara*	No applicable	South China Sea	Cytotoxicity against HeLa, A549, MDA231 and QGY7703 cells	Jiao et al., 2011 [46]
Dysidavarones B–C (**105–106**)	*Dysidea avara*	No applicable	South China Sea	No data	-
Siphonodictyal A sulfate (**107**)<br>Akadisulfates A–B (**108–109**)	*Aka coralliphaga*	No applicable	Mexico	Radical-scavenging activity	Shubina et al., 2012 [48]
6′-Iodoaureol (**110**)<br>6′-Aureoxyaureol (**111**)	*Smenospongia* sp.	No applicable	Thai	Cytotoxicity	Prawat et al., 2012 [49]
(-)-*N*-methylmelemeleone-A (**112**)	*Dysidea avara*	No applicable	Mediterranean Sea, Turkey	Cytotoxicity	Hamed et al., 2013 [50]
Dysideanones A–C (**113–115**)	*Dysidea avara*	No applicable	South China Sea	No data	Jiao et al., 2014 [51]
Siphonodictyals E1–E2 (**116–117**)	*Aka coralliphagum*	No applicable	San Salvador, Bahamas	No data	Gothel et al., 2014 [52]
Siphonodictyal E3 (**118**)	*Aka coralliphagum*	No applicable	San Salvador, Bahamas	Antibacterial against G^+	-
Siphonodictyal E4 (**119**)	*Aka coralliphagum*	No applicable	San Salvador, Bahamas	Antiproliferation against L929	-
Avapyran (**120**)<br>17-*O*-acetylavarol (**121**)<br>17-*O*-acetylneoavarol (**122**)	*Dysidea* sp.	No applicable	Okinawan, Japan	PTP1B inhibition	Abdjul et al., 2016 [53]
Langcoquinones A–B (**123-124**)	*Spongia* sp.	No applicable	Vietnam	Antibacterial	Nguyen et al., 2016 [54]

Dysidphenols A–C (**125**–**127**) Smenospongimine (**128**)	*Dysidea* sp.	No applicable	South China Sea	Antibacterial	Zhang et al., 2016 [55]
18-Deoxy-18-formamidodictyoceratin B (**129**) 18-Deoxy-18-(2-hydroxyacetyl) aminodictyoceratin B (**130**) Dictyoceratin D (**131**) *N*-Methyl-ent-smenospongine (**132**) *N*-Methyl-5-*epi*-smenospongine (**133**) 20-Demethoxy-20-methylamino-5-*epi*-dactyloquinone D (**134**) 20-Demethoxy-20-methylaminodactyloquinone B (**135**) Yahazunol B (**136**)	*Spongia pertusa* Esper	No applicable	Yongxing Island, China	No data	Li et al., 2017 [56]
20-Demethoxy-20-methylaminodactyloquinone D (137)	*Spongia pertusa* Esper	No applicable	Yongxing Island, China	CDK-2 affinity	-
Neoisosmenospongine (**138**)	*Dactylospongia metachromia*	No applicable	Bajotalawaan, Sulawesi	No data	Hitora et al., 2021 [57]
Xishaeleganins A, C–D (**139**, **141**–**142**)	*Dactylospongia elegans*	No applicable	Xisha Island, China	No data	Chen et al., 2022 [58]
Xishaeleganin B (**140**)	*Dactylospongia elegans*	No applicable	Xisha Island, China	Antibacterial activity	-
Dysideanones F–G (**143**–**144**)	*Dysidea avara*	No applicable	South China Sea	No data	Liu et al., 2022 [59]
Dysiherbols D–E (**145**–**146**)	*Dysidea avara*	No applicable	South China Sea	Anti-inflammatory	-
Arenarialins A–F (**147**–**152**)	*Dysidea arenaria*	No applicable	South China Sea	Anti-inflammatory	Li et al., 2024 [60]
Nakijiquinone S (**153**) Nakijinol C (**154**)	*Spongiidae* sp.	No applicable	Okinawan, Japan	Antimicrobial	Suzuki et al., 2014 [61]
(-)-Agelasine D (**155**) (-)-Ageloxime D (**156**)	*Agelas nakamurai*	No applicable	Menjangan Island, Indonesia	Antifouling, antibacterial, biofilm formation inhibition	Hertiani et al., 2010 [62]
18-Aminoarenarone (**157**) 19-Aminoarenarone (**158**) 18-Methylaminoarenarone (**159**) 19-Methylaminoarenarone (**160**)	*Dysidea* sp.	No applicable	Australia	No data	Utkina et al., 2010 [63]
Popolohuanone F (**161**)	*Dysidea* sp.	No applicable	Australia	DPPH radical scavenging activity	-
Tauroarenarones A–B (**162**–**163**)	*Dysidea* sp.	Fungus *Trichoderma* sp. TPU1237	Australia	PTP1B inhibition	Utkina et al., 2014 [64]

Dysidinoid A (**164**)	*Dysidea* sp.	No applicable	South China Sea	Antibacterial against MRSA	Jiao et al., 2014 [65]
Dysiherbols A–C (**165–167**)	*Dysidea* sp.	No applicable	Xisha Islands, China	NF-*κ*B inhibitory and cytotoxic	Jiao et al., 2012 [66]
Dysideanone E (**168**)	*Dysidea* sp.	No applicable	Xisha Islands, China	No data	-
Dihydroxybergamotene (**169**)<br>Chlorocylindrocarpol (**170**)<br>Acremofuranones A–B (**171–172**)	*Stelletta* sp. J05B-1	*Acremonium* sp.	Jeju Island, Korea	No data	Zhang et al., 2009 [67]
Isopyrodysinoic acid (**173**)<br>13-Hydroisopyrodysinoic acid (**174**)<br>Pyrodysinoic acid B (**175**)	*Dysidea robusta*	No applicable	Brazil	No data	Williams et al., 2009 [68]
Dysifragilisins A–B (**176–177**)	*Dysidea fragilis*	No applicable	Hainan, China	PTP1B inhibition and cytotoxicity	Yu et al., 2009 [69]
Negombatoperoxides B–D (**178–180**)<br>Negombatolactone (**181**)	*Negombata corticata*	No applicable	Formosa	No data	Chao et al., 2010 [70]
Nakijinol B (**182**)<br>Nakijinol B diacetate (**183**)<br>Smenospongines B–C (**184–185**)	*Dactylospongia elegans*	No applicable	Pugh Shoal, Truant Island	Inhibition against SF-268, H460, MCF-7, and HT-29 cells	Ovenden et al., 2011 [71]
Metachromins U–W (**186–188**)	*Thorecta reticulata*	No applicable	Hunter Island, Australia	Inhibition against SF-268, H460, MCF-7, and HT-29 cells	Ovenden et al., 2011 [72]
Halichonadins G–J (**189–192**)	*Halichondria* sp.	No applicable	Unten Port, Okinawa	No data	Suto et al., 2011 [73]
5-Chloroacremine A (**193**)<br>5-Chloroacremine H (**194**)<br>Acremines O–R (**195–198**)	*Anomoianthella rubra*	Fungus *Acremonium persicinum*	Mooloolaba, Queensland	No data	Suciati et al., 2013 [74]
(4*R*,5*R*)-muurol-1(6),10(14)-diene-4,5-diol (**199**)<br>(4*R*,5*R*)-muurol-1(6)-ene-4,5-diol (**200**)<br>(4*R*,5*R*,10*R*)-10-methoxymuurol-1(6)-ene-4,5-diol (**201**)<br>(4*S*)-4-hydroxy-1,10-seco-muurol-5-ene-1,10-dione (**202**)<br>(4*R*)-4-hydroxy-1,10-seco-muurol-5-ene-1,10-dione (**203**)<br>(6*S*,10*S*)-6,10-dihydroxy-7,8-seco-2,8-cyclo-muurol-4(5),7(11)-diene-12-oic acid (**204**)<br>(6*R*,10*S*)-6,10-dihydroxy-7,8-seco-2,8-cyclo-muurol-4(5),7(11)-diene-12-oic acid (**205**)	*Dysidea cinerea*	No applicable	Red Sea	No data	Kiem et al., 2014 [75]

Euryspongins A–C (**206–208**)	*Euryspongia* sp.	No applicable	Iriomote Island, Japan	No data	Yamazaki et al., 2013 [76] Yamazaki et al., 2015 [77]
Compounds **209, 212**	*Ircinia* sp.	No applicable	Korea	PPARδ agonistic activity	Hahn et al., 2014 [78]
Compounds **210–211, 213**	*Ircinia* sp.	No applicable	Korea	No data	-
Punctaporonins H–M (**214–219**)	*Niphates* sp.	Fungus *Hansfordia sinuosae*	South China Sea	Cytotoxic, antibacterial	Wu et al., 2014 [79]
Adametacorenol A (**220**)	Undescribed	Fungus *Penicillium adametzioides* AS-53	Hainan Island, South China Sea	No data	Liu et al., 2015 [80]
Adametacorenol B (**221**)	Undescribed	Fungus *Penicillium adametzioides* AS-53	Hainan Island, South China Sea	Cytotoxic against NCI-H446 cell line	-
Axiriabilines A–D (**222–225**)	*Axinyssa variabilis*	No applicable	South China Sea	No data	Li et al., 2017 [81]
6β-acetyl-4β,5β-dimethyl-1(10)-α-epoxy-7-oxodecalin (**226**)	*Carteriospongia foliascens*	No applicable	South China Sea	No data	Wu, 2011 [82]
Lamellodysidines A–B (**227–228**) *O*, *O*-Dimethyllingshuiolide A (**229**) 11-*epi*-*O*, *O*-Dimethyllingshuiolide A (**230**)	*Lamellodysidea herbacea*	No applicable	Indonesia	No data	Torii et al., 2017 [83]
Dysidealactams A–F (**231–236**) Dysidealactones A–B (**237–238**)	*Dysidea* sp. CMB-01171	No applicable	Australia	No data	Khushi et al., 2020 [84]
Copteremophilanes A–J (**239–248**)	*Xestospongia testudinaria*	Fungus *Penicillium copticola*	Weizhou Island	No data	Zhang et al., 2022 [85]
Bicyclolamellolactone A (**249**)	*Lamellodysidea* sp.	No applicable	Indonesia	BMP-induced alkaline phosphatase activity	Ohte et al., 2021 [86]
Diplopuupehenone (**250**)	*Dysidea* sp.	No applicable	America	DPPH radical scavenging activity	Utkina et al., 2011 [87]
Puupehenol (**251**)	*Dactylospongia* sp.	No applicable	Maui Island, Hawaii	Antioxidant and antimicrobial	Hagiwara et al., 2015 [88]
Asperaculin A (**252**)	*Xesto-spongia testudinaria*	Fungus *Aspergillus aculeatus* CRI323-04	Phi Phi Island, Thailand	No data	Ingavat et al., 2011 [89]
(+)-Spongiterpene (**253**) (-)-Spongiterpene (**254**)	*Spongia* sp.	No applicable	Zhanjiang, China	No data	Liang et al., 2021 [90]
Sponalisolides A–B (**255–256**)	*Spongia officinalis*	No applicable	Beihai, China	QS inhibition	Sun et al., 2018 [91]
Myrmekiones A–C (**257–259**)	*Myrmekioderma* sp.	No applicable	South China Sea	No data	Zhou et al., 2022 [92]

Ximaocavernosins A–O (**260–274**)	*Acanthella cavernosa*	No applicable	South China Sea	No data	Shen et al., 2022 [93]
Ximaocavernosin P (**275**) (+)-Maninsigin D (**276**) (+)- and (-)-Ximaocavernosin Q (**277–278**)	*Acanthella cavernosa*	No applicable	South China Sea	No data	Shen et al., 2022 [94]
Insulicolide D (**279**)	Unidentified	Fungus *Aspergillus insulicola* HDN151418	Antarctica	Suppress cell proliferation	Sun et al., 2022 [95-96]
Insulicolides E–G (**280–282**)	Unidentified	Fungus *Aspergillus insulicola* HDN151418	Antarctica	No data	-
Odoripenoids A–B (**283–284**)	*Spongia* sp.	Fungus *Streptomyces* sp. NBU3428	Xisha Island, China	Anti-*Candida albicans*	Wen et al., 2023 [97]
Odoripenoids C–D (**285–286**)	*Spongia* sp.	Fungus *Streptomyces* sp. NBU3428	Xisha Island, China	No data	-
Acremosides A, C–E (**287, 289–291**)	*Haliclona* sp.	Fungus *Acremonium* sp. IMB18-086	Weizhou Island, China	Inhibitory activities against hepatitis C virus	Hao et al., 2024 [98]
Acremosides B, F–G (**288, 292–293**)	*Haliclona* sp.	Fungus *Acremonium* sp. IMB18-086	Weizhou Island, China	No data	-
Cpd-8 (**294**)	*Tedania* sp.	Fungus *Aspergillus ochraceopetaliformis*	Penghu, East China Sea	Cytotoxicity	He et al., 2024 [99]
Smenohaimiens A–E (**295–299**)	*Smenospongia cerebriformis*	No applicable	Vietnam	Inhibition of NO production	Kiem et al., 2017 [100]
Quintaquinone (**300**) 5-*Epi*-Nakijiquinone L (**301**) Compound **302**	*Verongula* cf. *rigida*	No applicable	Thailand	No data	Jiso et al., 2020 [101]
(+)-19-methylaminoavarone (**303**)	*Dysidea* sp.	No applicable	Xisha Islands	Cytotoxicity	Luo et al., 2021 [102]
Purpurols A–D (**304–307**) Puraminones A–J (**308–317**)	*Pseudoceratina purpurea*	No applicable	South China Sea	Anti-inflammation for purpurols A–B	Yu et al., 2024 [103]

Table S2. Diterpenes isolated from the marine sponges.

Compound	Sponge Species	Sponge-Derived Microbe	Sampling Location	Biological Activity	Ref.
8-isocyanoamphilecta-11(20),15-diene (**318**)	*Ciocalapata* sp.	No applicable	Koh-Tao, Thailand	Antimalarial	Wattanapiromsakul et al., 2009 [104]
8-isocyanato-15-formamidoamphilect-11(20)-ene (**319**) 8-isothiocyanato-15-formamidoamphilect-11(20)-ene (**320**)	*Stylissa* cf. *massa*	No applicable	Koh-Tao, Thailand	Antimalarial	Chanthathamrongsiri et al., 2012 [105]
Chromodorolide D (**321**) Compound **322**	Unidentified	No applicable	Okinawa	Cytotoxicity against NBT-T2	Uddin et al., 2012 [106]
Compounds **323–326**	*Dysidea* cf. *Arenaria*	No applicable	Irabu Island, Okinawa	Cytotoxicity against NBT-T2 cell	Shingaki et al., 2016 [107]

Darwinolide **(327)**	*Dendrilla membranosa*	No applicable	Antarctica	Cytotoxicity against J774 macrophage cell and antibacterial	Salm et al., 2016 [108]
Oxeatine **(328)** Oxeatamides H–J **(329–331)**	*Darwinella* cf. *oxeata*	No applicable	Brazil	No data	Ramirez et al., 2017 [109]
Ceylonins A–F **(332–337)**	*Spongia ceylonensis*	No applicable	Indonesia	Partial inhibited osteoclastogenesis	El-Desoky et al., 2017 [110]
Ceylonins G–I **(338–340)**	*Spongia ceylonensis*	No applicable	Indonesia	No data	El-Desoky et al., 2017 [111]
Ceylonamides A–F **(341–346)** 15*α*,16-dimethoxyspongi-13-en-19-oic acid (**347**)	*Spongia ceylonensis*	No applicable	Indonesia	Two of them inhibited osteoclastogenesis	El-Desoky et al., 2016 [112]
3-Nor-spongiolide A (**348**) Spongiolides A–B (**349-350**)	*Spongia officinalis*	No applicable	South China Sea	No data	Han et al., 2018 [113]
3*β*-acetoxy-15-hydroxyspongia-12-ene (**351**) 3-methylspongia-3,12-dien-16-one (**352**)	*Acanthodendrilla* sp.	No applicable	Pulau	No data	Costa et al., 2020 [114]
Dendrillin B (**353**)	*Dendrilla antarctica*	No applicable	Antarctica	Anti-leishmaniasis parasite	Bory et al., 2020 [115]
Dendrillin C **(354)**	*Dendrilla antarctica*	No applicable	Antarctica	Anti-MRSA	-
Dendrillin D (**355**)	*Dendrilla antarctica*	No applicable	Antarctica	No data	-
Spongenolactones A–C (**356–358**)	*Spongia* sp.	No applicable	Red Sea	Inhibit O^{-} generate and antimicrobial	Tai et al., 2022 [116]
Spongionellol A (**359**)	*Spongionella* sp.	No applicable	Gulf, Sakhalin	Cytotoxicity against human prostate cancer cell	Dyshlovoy et al., 2022 [117]
2*β*,3*α*,19-Triacetoxy-17-hydroxyspongia-13(16),14-diene (**360**)	*Spongia officinalis*	No applicable	Hainan, China	Cytotoxicity against K562	Jin et al., 2023 [118]
18-nor-2,17-hydroxyspongia-1,4,13(16),14-quaien-3-one (**361**)	*Spongia officinalis*	No applicable	Hainan, China	No data	-
Secodinorspongin A (**362**)	*Spongia* sp.	No applicable	Red Sea	Antimicrobial against *S. aureus*.	Tai et al., 2023 [119]
Secodinorspongins B–D (**363–365**)	*Spongia* sp.	No applicable	Red Sea	No data	-
Dendrillic acid A **(366)**	*Dendrilla* sp.	No applicable	Y-Island, Exmouth Gulf	No data	Sala et al., 2023 [120]
Dendrillic acid B **(367)**	*Dendrilla* sp.	No applicable	Y-Island, Exmouth Gulf	Antiprotozoal activity	-
3*β*-hydroxyspongia-13(16),14-dien-2-one (**368**) 19-dehydroxy-spongian diterpene 17 (**369**)	*Spongia tubulifera*	No applicable	Mexican Caribbean	Cytotoxic for **368**	Pech-Puch et al., 2019 [121]
Isospongiatriol (**370**) 3-*nor*-Spongianone A (**371**)	*Spongia* sp.	No applicable	Fiji Island	No data	Gross et al., 2009 [122]

3-*nor*-Spongianone B (**372**)<br>Ircinolin A (**373**)<br>10- Acetylirciformonin B (**375**)	*Ircinia* sp.	No applicable	Orchid Island, Taiwan	Cytotoxicity	Su et al., 2011 [123]
Acetylirciformonin B (**374**)	*Ircinia* sp.	No applicable	Orchid Island, Taiwan	No data	-
7, 8-Epoxyfurospongin-1 (**376**)	*Spongia officinalis*	No applicable	Mediterranean	No data	Manzo et al., 2011 [124]
Hamigerans F–L (**377–383**)<br>10-*epi*-hamigeran K (**384**)<br>4-Bromohamigeran K (**385**)<br>Hamigeran L 11-*O*-methyl ester (**386**)	*Hamigera tarangaensis*	No applicable	New Zealand	Cytotoxicity against HL-60 and antifungal for Hamigeran G	Singh et al., 2013 [125]
Hamigeran A ethyl ester (**387**)<br>Hamigerans R- S (**388-389**)4-Bromohamigeran A (**390**)<br>Debromohamigerans B, I–J (**391–393**)<br>Hamigeran L 12-*O*-methyl ester (**394**)	*Hamigera tarangaensis*	No applicable	New Zealand	Cytotoxicity against HL-60 cells	Woolly et al., 2018 [126]
Hamigerans M-N, O-P, Q (**395–396, 398–399, 401**)<br>**18-***epi*-hamigerans N, P, Q (**397, 400, 403**)<br>19-*epi*-hamigeran Q (**402**)	*Hamigera tarangaensis*	No applicable	Cavalli Island, New Zealand	Cytotoxicity against HL-60 cell	Dattelbaum et al., 2015 [127]
Oxeatamide A (**404**)<br>iso-Oxeatamide A (**405**)<br>Oxeatamides B–G (**406–411**)<br>Oxeatamide A 23-methyl ester (**412**)	*Darwinella oxeata*	No applicable	New Zealand	No data	Wojnar et al., 2014 [128]
Luakuliide A (**413**)<br>Luakuliide A methyl seter (**414**)	*Hamigera tarangaensis*	No applicable	Eua Island and Vava'u Island, Tonga	Cytotoxicity against HL-60 cells	Barber et al., 2015 [129]
Luakuliides B–C (**415–416**)	*Hamigera tarangaensis*	No applicable	Eua Island and Vava'u Island, Tonga	No data	-
Amitorines A–B (**417–418**)	*Theonella swinhoei*	No applicable	Iriomote Island, Japan	No data	Ota et al., 2016 [130]
Sponalactone (**419**)<br>17-*O*-acetylepispongiatriol (**420**)<br>17-*O*-acetylspongiatriol (**421**)	*Spongia officinalis*	No applicable	South China Sea	Inhibition against LPS-induced NO production	Chen et al., 2019 [131]
Clavrolide H (**422**)	*Fascaplysinopsis reticulata*	No applicable	Xisha Islands, China	No data	Qin et al., 2020 [132]
6,10,18-triacetoxy-2*E*,7*E*-dolabelladiene (**423**)	*Luffariella variabilis*	No applicable	South China Sea	No data	Luo et al., 2021 [133]
Kalihioxepanes A–B (**424–425**)	*A. cavernosa*	No applicable	South China Sea	Cytotoxicity against H69 and K562	Wang et al., 2022 [134]
Kalihioxepanes C–G (**426–430**)	*A. cavernosa*	No applicable	South China Sea	No data	-

Kalihiacyloxyamides A–H (**431**–**438**)	*Acanthella cavernosa*	No applicable	South China Sea	Cytotoxicity for four of them	Wang et al., 2023 [135]
Tricycloalternarenes A, B–C (**439**, **446**–**447**) Bicycloalternarenes A–F (**440**–**445**) Monocycloalternarenes A–D (**448**–**451**)	*Callyspongia* sp.	Fungus of *Alternaria* sp. JJYNo applicable32	Hainan Island, China	NF-κB Inhibition	Zhang et al., 2013 [136]
Chartarlactams A–C (**452**–**454**) Chartarlactams G–J (**458**–**461**) Chartarlactams M, P (**464**, **467**)	*Niphates recondite*	Fungus *Stachybotrys chartarum*	Weizhou Island, China	Lipid-lowering	Li et al., 2014 [137]
Chartarlactams D–F (**455**–**457**) Chartarlactams K–L (**462**–**463**) Chartarlactams N–O (**465**–**466**)	*Niphates recondite*	Fungus *Stachybotrys chartarum*	Weizhou Island, China	No data	-
(1*S*,3*S*,4*R*,7*S*,8*S*,11*S*,12*S*,13*S*,15*R*,20*R*)-7-Formamido-20-isocyanoisocycloamphilectane (**468**) (1*S*,3*S*,4*R*,7*S*,8*S*,11*S*,12*S*,13*S*,15*R*,20*R*)-7,20-Diformamidoisocycloamphilectane (**469**)	*Cymbastela hooperi*	No applicable	Tropical	Antiplasmodial	Wright et al., 2009 [138]
(1*S**,3*S**,4*R**,7*S**,8*S**,12*S**,13*S**)-7-Formamidocycloamphilect-11(20)-ene (**470**) (1*R**,3*S**,4*R**,7*S**,8*S**,12*S**,13*S**)-7-Formamidoamphilecta-11(20),14-diene (**471**) (1*S**,3*S**,4*R**,7*S**,8*S**,12*S**,13*S**)-7-Formamidoamphilecta-11(20),15-diene (**472**)	*Cymbastela hooperi*	No applicable	Tropical	No data	-
Gracilins J-L (**473**-**475**) 3'-norspongiolactone (**476**)	*Spongionella* sp.	No applicable	West Angaur, Philippines	Cytotoxic against K562	Rateb et al., 2009 [139]
7α,11α-diacetoxyisoagatholactone (**477**) Compounds **478**–**479** 11β-acetoxyspongian-13-en-16-one (**480**)	*Dysidea* cf. *arenaria*	No applicable	Okinawa	Cytotoxicity against NBT-T2	Agena et al., 2009 [140]
Agelasine M (**481**) 2-*oxo*-Agelasine B (**482**) Gelasine A (**483**)	*Agelas* sp.	No applicable	Papua New Guinea	Cytotoxicity against *Jurkat* cells and inhibition against *T. brucei*	Calcul et al., 2010 [141]
Gelasine B (**484**)	*Agelas* sp.	No applicable	Papua New Guinea	No data	-
Negombatodiol (**485**)	*Negombata corticata*	No applicable	Formosa	No data	Chao et al., 2010 [70]
Clathric acid **(486)**	*Clathria compressa*	No applicable	Panama, Florida	Antibacterial activity	Gupta et al., 2012 [142]
Clathrimides A–B (**487**–**488**)	*Clathria compressa*	No applicable	Panama, Florida	No data	-

Tetradehydrofurospongin-1 epoxylactone (**489**)	*Coscinoderma matthewsi*	No applicable	Inner Gneerings Reef, Queensland	No data	Katavic et al., 2012 [143]
Chromodorolides D–E (**490–491**)	*Dysidea* sp.	No applicable	Inner Gneerings Reef, Queensland	No data	-
(±)-8,13-secoepicavernosinei (**492**) Cacospongine A (**493**)	*Cacospongia* sp.	No applicable	South China Sea	No data	Zhang et al., 2018 [144]
Axistatins 1–3 **(494–496)**	*Agelas axifera* Hentschel	No applicable	Republic of Palau	Cytotoxicity against cancer cells and antimicrobial	Pettie et al., 2013 [145]
Gukulenins C–F (**497–500**)	*Phorbas gukhulensis*	No applicable	Gagu-do, Korea	Cytotoxicity against K562 and A549 cells	Jeon et al., 2013 [146]
Halioxepine (**501**)	*Haliclona* sp.	No applicable	Indonesia	Cytotoxicity against NBT-T2, antioxidant	Trianto et al., 2011 [147]
Xestolactones A, O **(502, 503)**	*Xestospongia vansoesti*	No applicable	Philippines	No data	Centko et al., 2014 [148]
Spiroarthrinols A–B (**504–505**)	*Sarcotragus muscarum*	Fungus *Arthrinium* sp.	Turkey	No data	Elissawy et a., 2017 [149]
19-Norspongia-13(16),14-diene-3-one (**506**)	*Spongia*	No applicable	Suwarrow atoll, Pacific Ocean	Inhibited sea urchin embryo development and DNA biosynthesis	Ponomarenko et al., 2011 [150]
Arthrinins A–D (**507–510**)	*Geodia cydonium*	Fungus *Arthrinium* sp.	Mediterranean	No data	Ebada et al., 2011 [151]
Myrocin D (**511**)	*Geodia cydonium*	Fungus *Arthrinium* sp.	Mediterranean	Inhibited VEGF-A cell	-
Emindole SB *beta*-mannoside **(512)**	*Callyspongia* sp. cf. C. *flammea*	Fungus *Dichotomomyces cejpii*	Bear Island, Australia	CB2 antagonist	Harms et al., 2014 [152]
27-*O*-methylasporyzin C **(513)**	*Callyspongia* sp. cf. C. *flammea*	Fungus *Dichotomomyces cejpii*	Bear Island, Australia	Selective GPR18 antagonist	-
18-nor-3,17-dihydroxyspongia-3,13(16),14-trien-2-one (**514**) 18-nor-3,5,17-trihydroxyspongia-3,13(16),14-trien-2-one (**515**) Spongiapyridine **(516)**	*Spongia* sp.	No applicable	Bunaken Marine Park, Indonesia	Partial inhibited aromatase and induced quinone reductase 1	Parrish et al., 2014 [153]
26-*O*-ethylstrongylophorine-14 **(517)**	*Strongylophora strongilata*	No applicable	Iriomote Island, Japan	Inhibited PTP1B	Lee et al., 2015 [154]
26-O-methylstrongylophprine-16 **(518)** 26-O-ethylstrongylophorine-16 **(519)**	*Petrosia corticata*	No applicable	Sulawesi, Indonesia	Proteasome inhibitors	Noda et al., 2015 [155]

Niphateolide A (**520**)	*Niphates olemda*	No applicable	Indonesia	p53-Hdm2 interaction inhibitor	Kato et al., 2015 [156]
Stachybotrin G (**521**)	*Xestospongia testudinaria*	Fungus *Stachybotrys chartarum* (MXH-X73)	Xisha Island, China	No data	Ma et al., 2015 [157]
Rhabdaprovidines A–C (**522–524)**	*Rhabdastrella providentiae*	No applicable	Vietnam	NO inhibition	Dung et al., 2018 [158]
Dysiarenone (**525**)	*Dysidea arenaria*	No applicable	South China Sea	COX-2 and PG-E2 inhibition	Jiao et al., 2018 [159]
Cyclobutastellettolides A–B **(526–527)**	*Stelletta* sp.	No applicable	Vietnam	Increase the reactive oxygen	Kolesnikova et al., 2019 [160]
Septosone A **(528)**	*Dysidea septosa*	No applicable	South China Sea	Anti-inflammatory	Gui et al., 2019 [161]
Septosones B–C (**529–530)**	*Dysidea septosa*	No applicable	South China Sea	No data	-
(–)-agelamide D **(531)**	*Agelas* sp.	No applicable	Chuuk, Micronesia	Radiosensitizer	Choi et al., 2020 [162]
Stellettins S–V **(532–535)**	*Stelletta* sp.	No applicable	Vietnam	No data	Kolesnikova et al. 2021 [163]
Compounds **536–538**	*Astrosclera willeyana*	No applicable	Tonga	No data	Jiang et al., 2021 [164]
Ascandinines A–B (**539–540**)	Unidentified	Fungus *Aspergillus candidus* HDN15-152	Pulitzer Bay, Antarctica	No data	Zhou et al., 2021 [165]
Ascandinine C **(541)**	Unidentified	Fungus *Aspergillus candidus* HDN15-152	Pulitzer Bay, Antarctica	Anti-influenza virus A	-
Ascandinine D (**542**)	Unidentified	Fungus *Aspergillus candidus* HDN15-152	Pulitzer Bay, Antarctica	Cytotoxicity against HL-60	-
17-dehydroxysponalactone **(543)**	*Spongia* sp.	No applicable	Red Sea	No data	Tai et al., 2021 [166]
Guignardone Y (**544**)	*Crella*	Fungus *Penicillium* sp. NBUF154	No data	Inhibitory against EV71	Zou et al., 2022 [167]
Guignardone Z (**545**)	*Crella*	Fungus *Penicillium* sp. NBUF154	No data	No data	-
(+)-8-epiagelasine T (**546**)<br>(+)-10-epiagelasine B (**547**)<br>(+)-12-hydroxyagelasidine C (**548**)	*Agelas citrina*	No applicable	Yucatán Peninsula, Mexico	Antimicrobial activity	Pech-Puch et al., 2022 [168]
Compounds **549–551**	*Diacarnus spinipoculum*	No applicable	Bohol Island, Philippines	No data	Cho et al., 2024 [169]
9,11-Dihydrogracillinone A (**552**)	*Dendrilla antarctica*	No applicable	Puerto Deseado	Antifouling activity	Prieto et al. 2022 [170]
Sanyanolides C, E–F, G–I (**553–558**)	*Chelonaplysilla* sp.	No applicable	South China Sea	No data	Shen et al., 2023 [171]
Echinohalimane B (**559**)<br>Oculatolide B **(560)**	*Sarcotragus* sp.	No applicable	South China Sea	No data	Xu et al., 2023 [172]
Pestanoid A **(561)**	*Chalinidae* sp.	Fungus *Pestalotiopsis* sp. NBUF145	Paracel Islands, Hainan, China	Inhibited bone marrow monocyte osteoclastogenesis	Wang et al., 2024 [173]

Table S3. Sesterterpenes isolated from the marine sponges.

Compound	Sponge Species	Sponge-Derived Microbe	Sampling Location	Biological Activity	Ref.
Coscinolactams A–B (**562–563**)	*Coscinoderma mathewsi*	No applicable	Formosa	Anti-inflammatory	De Marino et al., 2010 [174]
Phyllofolactone D (**564**)	*Phyllospongia foliascens*	No applicable	South China Sea	Cytotoxic against the P388 leukemia cell	Zhang et al., 2010 [175]
Phyllofolactone L (**565**)<br>Phyllofolactone E (**566**)	*Phyllospongia foliascens*	No applicable	South China Sea	No data	-
Phyllofolactone M (**567**)	*Phyllospongia foliascens*	No applicable	South China Sea	No data	Zhang et al., 2010 [176]
Similan A (**568**)<br>12beta,20-dihydroxy-16beta-acetoxy-17-scalaren-19,20-olide (**569**)<br>12beta-acetoxy-20-hydroxy-17-scalaren-19,20-olide (**570**)<br>12beta,16alpha,20-trihydroxy-17-scalaren-19,20-olide (**571**)	*Hyrtios gumminae*	No applicable	Thailand	No data	Mahidol et al., 2009 [177]
Compounds **572–575**	*Carteriospongia foliascens*	No applicable	Makassar, Indonesia	Cytotoxicity and Partial RCE-Protease inhibition	Williams et al., 2009 [178]
Compounds **576–588** (except **579**)<br>Phyllofenone C (**589**)	*Carteriospongia foliascens*	No applicable	South China Sea	No data	Wu, 2011 [82]
Compound **579**	*Carteriospongia foliascens*	No applicable	South China Sea	Antifouling activity	-
Compound **590**	*Carteriospongia foliascens*	No applicable	South China Sea	Toxicity	-
Compounds **591–595**	*Hyatella* sp.	No applicable	Soheuksando, Korea	Cytotoxicity, antibacterial activity	Jeon et al., 2011 [180]
12-deacetoxy-23-hydroxyscalaradial (**596**)<br>12-dehydroxy-23-hydroxyhyrtiolide (**597**)<br>12-O-acetyl-16-deacetoxy-23-acetoxyscalarafuran (**598**)	*Psammocinia sp.*	No applicable	Korea	Cytotoxicity	Hahn et al., 2013 [181]
16-*epi*-Scalarafuran **(599)**<br>Petrosaspongiolactams B-C **(602–603)**	*Hyrtios* sp. and *Petrosaspongia* sp.	No applicable	Fiji Islands	No data	Festa et al., 2014 [182]
Compound **600**<br>Petrosaspongiolactam A **(601)**	*Hyrtios* sp. and *Petrosaspongia* sp.	No applicable	Fiji Islands	Inhibited the TDP-43 protein	-
Scalarinether **(604)**<br>21-hydroxy-16-deacetyl-12-epi-scalarafuran acetate (**605**)	*Hyrtios erectus*	No applicable	South China Sea	No data	Lu et al., 2014 [183]

Phyllospongins A–E (**606–610**)	*Phyllospongia lamellose*	No applicable	Red Sea	Cytotoxicity and antibacterial activity	Hassan et al., 2015 [184]
Lendenfeldaranes E–G (**611–613**)	*Lendenfeldia* sp.	No applicable	Taiwan, China	Anti-neutrophilic	Peng et al., 2020 [185]
Lendenfeldaranes H–J (**614–616**)	*Lendenfeldia* sp.	No applicable	Taiwan, China	No data	-
Compounds **617–622, 624–631**	*Dysidea* sp.	No applicable	Bohol, Philippines	No data	Shin et al., 2021 [186]
Compound **623**	*Dysidea* sp.	No applicable	Bohol, Philippines	Cytotoxicity	-
Dysiscalarones A–B (**632–633**)	*Dysidea granulosa*	No applicable	South China Sea	NO production inhibition	Sun et al., 2021 [187]
Dysiscalarones C–E (**634–636**)	*Dysidea granulosa*	No applicable	South China Sea	No data	-
lendenfeldaranes K–M (**637–639**)	*Lendenfeldia* sp.	No applicable	Taiwan, China	Anti-inflammatory	Peng et al., 2021 [188]
lendenfeldaranes N–Q (**640–643**)	*Lendenfeldia* sp.	No applicable	Taiwan, China	No data	-
Hyrtioscalaranes A–B (**644–645**)	*Hyrtios erectus*	No applicable	Arabian Sea	Anti-inflammatory activities and antioxidant activities	Chakraborty and Francis, 2021 [189]
12*β*,20*β*-Dihydroxy-16*α*-methoxy-17-scalaren-19,20-olide (**646**)<br>12*β*,20*α*-Dihydroxy-16*β*-methoxy-17-scalaren-19,20-olide (**647**)<br>12*β*,16*β*,20*β*-Trihydroxy-17-scalaren-19,20-olide (**648**)<br>12*β*,19*α*(*β*)-Dihydroxy-16*α*-methoxy-17-scalaren-19,20-olide (**649**)<br>12*β*,19*α*(*β*)-Dihydroxy-16*β*-methoxy-17-scalaren-19,20-olide (**650**)<br>12*β*,19*α*-Dihydroxy-14,15-dehydrate-17-scalaren-19,20-olide (**651**)<br>12-Deacetyl-18-*epi*-carboxylic-12-*epi*-scalaral (**652**)<br>2-O-Deacetyl-12,16-di-*epi*-norscalaral B (**653**)	*Hyrtios erectus*	No applicable	Bohol Island, Philippines	Cytotoxicity	Tran et al., 2022 [190]
Phyllofenones F–M (**654–661**)	*Phyllospongia foliascens*	No applicable	South China Sea	Cytotoxicity, antibacterial for **659** and **661**	Yu et al., 2023 [191]
Phyllospongianes A–B, D (**662–663, 665**)	*Phyllospongia foliascens*	No applicable	Woody Island, South China Sea	Antibacterial activity	Yu et al., 2023 [192]
Phyllospongiane C (**664**)	*Phyllospongia foliascens*	No applicable	Woody Island, South China Sea	Cytotoxic activity	-

Phyllospongiane E **(666)**	*Phyllospongia foliascens*	No applicable	Woody Island, South China Sea	No data	-
Hippospongides A–B (**667–668**)	*Hippospongia* sp.	No applicable	Taiwan, China	No data	Chang et al., 2012 [193]
Granulosane A (**669**) Compounds **670–682** (except **672**)	*Dysidea granulosa*	No applicable	South China Sea	No data	Wang et al., 2020 [194]
Compound **672**	*Dysidea granulosa*	No applicable	South China Sea	Antiproliferative activity	-
Scalarester (**683**)	*Dysidea* sp.	No applicable	South China Sea	No data	Yang et al., 2010 [195]
7*E*, 12*E*, 20*Z*-variabilin (**684**) Cavernosolide (**685**) Lintenolide A (**686**)	*Semitaspongia bactriana*	No applicable	New Zealand	Toxicity	Stewart et al., 2009 [196]
Alotaketals A–B (**687–688**)	*Hamigera* sp.	No applicable	Papua New Guinea	cAMP cell agonist	Forestieri et al., 2009 [197]
Ansellone A **(689)**	*Phorbas* sp.	No applicable	Howe Sound, Canada	cAMP cell agonist	Daoust et al., 2010 [198]
Ansellone B (**690**) Phorbadione (**691**) Secoepoxyansellone A (**692**)	*Phorbas* sp.	No applicable	British Columbia	No data	Daoust et al., 2013 [199]
Alotaketal C (**693**)	*Phorbas* sp.	No applicable	British Columbia	cAMP cell agonist	-
Phorbaketals A–C **(694–696)**	*Phorbas* sp.	No applicable	Korea	Cytotoxicity against human colorectal, hepatoma, lung cancer cells	Rho et al., 2009 [200]
Phorbaketals L–M **(697–698)**	*Phorbas* sp.	No applicable	Gageo Island, Korea	No data	Lee et al., 2014 [201]
Phorbaketal N **(699)**	*Phorbas* sp.	No applicable	Gageo Island, Korea	Cytotoxicity	-
Phorbaketals D–G, J–K **(700–703, 706–707)**	*Monanchora* sp.	No applicable	Korea	No data	Wang et al., 2013 [202]
Phorbaketals H–I **(704–705)** Phorbin A **(708)**	*Monanchora* sp.	No applicable	Korea	Cytotoxicity	-
Phorbasone A (**709**)	*Phorbas* sp.	No applicable	Korea	Positive effect on calcium deposition activity	Rho et al., 2011 [204]
Phorbasone B (**710**)	*Phorbas* sp.	No applicable	Korea	No data	-
Phorone A **(711)** Isophorbasone A **(712)**	*Phorbas* sp.	No applicable	Korea	No data	Wang et al., 2012 [205]
Irciformonins E–H, J–K (**713–716**, **718–719**)	*Ircinia formosana*	No applicable	Taiwan, China	No data	Shen et al., 2009 [206]
Irciformonins I (**717**)	*Ircinia formosana*	No applicable	Taiwan, China	Inhibited cell proliferation	-

Isofusospongin-4 (**720**)	*Spongia officinalis*	No applicable	Mediterranean	No data	Manzo et al., 2011 [124]
Ircinialactams E–F **(721–722)**	*Ircinia oros*	No applicable	Aegean Sea, Turkey	Antiprotozoal activity	Chianese et al., 2017 [207]
Sulawesins A–B **(723–724)**	*Psammocinia* sp.	No applicable	Sulawesi, Indonesia	USP7 inhibition	Afifi et al., 2017 [208]
Sulawesin C **(725)**	*Psammocinia* sp.	No applicable	Sulawesi, Indonesia	No data	-
(+)-Muqubilone B (**726**)	*Diacarnus bismarckensis*	No applicable	Sanaroa, Papua New Guinea	Inhibition against T. *brucei*	Rubio et al., 2009 [209]
(-)-Entmuqubilone B (**727**)	*Diacarnus bismarckensis*	No applicable	Sanaroa, Papua New Guinea	No data	-
(-)-13, 14-Epoxymuqubilin A **(728)**<br>(-)-9, 10-Epoxymuqubilin A **(729)**	*Diacarnus erythraeanus*	No applicable	Red Sea	Cytotoxicity	Lefranc et al., 2013 [210]
Megaspinoxide A (**730**)	*Diacarnus megaspinorhabdosa*	No applicable	No data	Antimicrobial against *Bacillus cereus*, *Staphylococcus aureus*, and *Candida albicans*	Ibrahim et al., 2014 [211]
Hippolides A–H (**731–738**)	*Hippospongia lachne*	No applicable	South China Sea	Partial cytotoxicity, PTP1B inhibition and anti-inflammatory activity	Piao et al., 2011 [212]
Compound **739**	*Coscinoderma* sp.	No applicable	Chuuk Island, Micronesia	No data	Bae et al., 2011 [213]
Compounds **740–746**	*Coscinoderma* sp.	No applicable	Chuuk Island, Micronesia	Cytotoxicity and enzyme inhibition	-
Terretonins E–F (**747–748**)	*Petrosia ficiformis*	Fungus *Aspergillus insuetus*	Mediterranean	NADH oxidase activity	López-Gresa et al., 2009 [214]
Fascioquinol A (**749**)	*Fasciospongia* sp.	No applicable	Southern Australia	Gram-positive selective antibacterial activity	Zhang et al., 2011 [215]
Fascioquinols E–F (**750–751**)	*Fasciospongia* sp.	No applicable	Southern Australia	No data	-
Austalides M–Q (**752–756**)	*Tethya aurantium*	Fungus *Aspergillus sp.*	Italy	No data	Zhou et al., 2011 [216]
Austalides S–T (**757–758**)	Undescribed	Fungus *Aspergillus aureolatus* HDN14-107	Xisha Islands, China	No data	Peng et al., 2016 [217]
Austalide U (**759**)	Undescribed	Fungus *Aspergillus aureolatus* HDN14-107	Xisha Islands, China	Anti-H1N1	-
Flabelliferins A–B (**760–761**)	*Cateriospongia flabellifera*	No applicable	Vanuatu	Growth inhibition activity	Diyabalanage et al., 2012 [218]

Thorectidaeolides A–B (**762**, **764**) 3-Acetoxythorectidaeolide A **(763)**	*Hyrtios communis*	No applicable	Palau	Inhibitors of hypoxia-induced HIF-1 activation	Li et al., 2013 [219]
Thorectidaeolides C–E (**765**–**767**)	*Hyrtios communis*	No applicable	Palau	No data	-
Compounds **768, 770–772**	*Hippospongia lachne*	No applicable	South China Sea	PTP1B inhibition for **768** and **772**, cytotoxicity for **768**	Piao et al., 2014 [220]
Compound **769**	*Hippospongia lachne*	No applicable	South China Sea	No data	-
Gombaspiroketals A, C **(773, 775)**	*Clathria gombawuiensis*	No applicable	Chuuk Island, Micronesia	Antibacterial activity, enzymes inhibition, and cytotoxicity against K562 and A549	- Woo et al., 2014 [221]
Gombaspiroketal B **(774)**	*Clathria gombawuiensis*	No applicable	Chuuk Island, Micronesia	No data	-
Chevalone E **(776)**	*Rhabdermia* sp.	Fungus *Aspergillus similanensis* KUFA 0013	Similan Islands, Thailand	Antimicrobial activity against MRSA	Prompanya et al., 2014 [222]
Hyattellalactones **A**–**B** **(777–778)**	*Hyattella* sp.	No applicable	Indonesia	PTP1B inhibition	Abdjul et al., 2014 [223]
Phorone B **(779)** Ansellone C **(780)**	*Clathria gombawuiensis*	No applicable	Korea	No data	Woo et al., 2015 [224]
Alotaketal D **(781)** Anvilone A **(787)**	*Phorbas* sp.	No applicable	Howe Sound British Columbia	Induce HIV gene expression	Wang et al., 2016 [225]
Alotaketal E **(782)** Ansellones D–G **(783–786)** Anvilone B **(788)**	*Phorbas* sp.	No applicable	Howe Sound British Columbia	No data	-
Hippolide J **(789)**	*Hippospongia lachne*	No applicable	South China Sea	Antifungal activity	Jiao et al., 2017 [226]
Balibaloside **(790)** 6''-O-acetylbalibaloside **(791)** 6'''-O-acetylbalibaloside **(792)** 6'', 6'''-O-acetylbalibaloside **(793)**	*Oscarella balibaloi*	No applicable	Mediterranean	No data	Audoin et al., 2013 [227]
(+)-hippolide E (**794**) (+)-(6*E*)-neomanoalide (**795**) (3*R*,4*R*)-14,18-secoluffariolide C (**796**)	*Cacospongia* sp.	No applicable	South China Sea	No data	Zhang et al., 2018 [144]
Oshimalides A–B (**797**–**798**)	*Luffariella* sp.	No applicable	Japan	No data	Kanki et al., 2021 [228]
Compounds **799**–**810**	*Luffariella variabilis*	No applicable	South China Sea	Cytotoxic activity	Luo et al., 2021 [229]

Dactylospenes A, C (**811, 813**)	*D. elegans*	No applicable	South China Sea	Cytotoxicity	Yu et al., 2020 [230]
Dactylospenes B, D–E (**812, 814–815**)	*D. elegans*	No applicable	South China Sea	No data	-
Sarcotragusolides A–D (**816–819**) Dactylospene F (**820**) 12-*β*-O-acetylhyrtiolide (**821**)	*Sarcotragus* sp.	No applicable	South China Sea	Cytotoxicity	Xu et al., 2023 [172]
Hippotulosas A–D (**822–825**)	*Hippospongia fistulosa*	No applicable	Vanphong Bay, Vietnam	No data	Hang et al., 2022 [231]
Neosuberitenone (**826**) Suberitenones E, G–J (**827, 829-832**) Secosuberitenone A (**833**) Norsuberitenone A (**834**)	*Suberites* sp.	No applicable	Antarctica	No data	Bracegirdle et al. 2023 [232]
Suberitenones F (**828**)	*Suberites* sp.	No applicable	Antarctica	Active against RSV	-
Isosuberitenone B **(835)** 19-episuberitenone B **(836)** Isooxaspirosuberitenone **(837)**	*Phorbas areolatus*	No applicable	Antarctica	Cytotoxicity	Solanki et al., 2018 [233]
Ircinianin lactones B-C (**838–839**)	*Ircinia wistarii*	No applicable	Wistari Reef, Australia	No data	Majer et al., 2022 [234]
19-Oxofasciospongine A (**840**) Fasciospongine C (**841**) 25-Hydroxyhalisulfate 9 (**842**)	*Fasciospongia* sp.	No applicable	Palau	Antibacteria, cytotoxic for **840**	Yao et al., 2009 [235]
Negombatoperoxide A (**843**)	*Negombata corticata*	No applicable	Formosa	No data	Chao et al., 2010 [70]
Insuetolide A (**844**)	*Psammocinia* sp.	Fungus *Aspergillus insuetus*	Israel	Anti-fungal against *Neurospora crassa*	Cohen et al., 2011 [21]
Insuetolide B (**845**)	*Psammocinia* sp.	Fungus *Aspergillus insuetus*	Israel	No data	-
Insuetolide C (**846**)	*Psammocinia* sp.	Fungus *Aspergillus insuetus*	Israel	Cytotoxicity towards MOLT-4 human leukemia cells	-
Stachybotrins D–F (**847–849**) Stachybocins E–F (**850–851**) Stachybosides A–B (**852–853**)	*Xestospongia testudinaris*	Fungus *Stachybotrys chartarum* MXH-X73	Xisha Island, China	Anti-HIV	Ma et al., 2013 [236]
Irciniasulfonates C–G **(854–858)**	*Coscinoderma* sp.	No applicable	Chuuk Island, Micronesia	Antibacterial activity, enzymes inhibition, and cytotoxicity against K562 and A549	Kim et al., 2014 [237]

Brasilianoid A (**859**)	Unidentified	Fungus *Penicillium brasilianum*	South China Sea	Stimulated the expression of filaggrin and caspase-14	Zhang et al., 2018 [238]
Brasilianoids B–C (**860–861**)	Unidentified	Fungus *Penicillium brasilianum*	South China Sea	Inhibition of NO production	-
Brasilianoids D–F (**862–864**)	Unidentified	Fungus *Penicillium brasilianum*	South China Sea	No data	-
Brasilianoids G–K (**865–869**)	Unidentified	Fungus *Penicillium brasilianum*	South China Sea	No data	Zhang et al., 2019 [239]
Brasilianoid L (**870**)	Unidentified	Fungus *Penicillium brasilianum*	South China Sea	Antibacterial	-
Dysivillosins A–D **(871–874)**	*Dysidea villosa*	No applicable	South China Sea	Anti-allergic	Jiao et al., 2016 [240]
Fintiamin (**875**)	*Ircinia variabilis*	Fungus *Eurotium* sp	No data	CB_1 receptor	Elsebai et al., 2021 [241]
Tandyukisins A–E **(876–880)**	*Halichondria okadai*	Fungus *Trichoderma harzianum* OUPS-111D-4	Osaka bay, Japan	Cytotoxicity against P388, HL-60 and LI210 cells	Suzue et al., 2016 [242] Takeshi et al., 2015 [282] Yamada et al., 2014 [243]
Tandyukisin F **(881)**	*Halichondria okadai*	Fungus *Trichoderma harzianum* OUPS-111D-4	Osaka bay, Japan	No data	-
16-*epi*-irciformonin G (**882**)	*Spongia* sp.	No applicable	Red Sea	No data	Tai et al., 2021 [166]
Hyrtamide A (**883**)	*Hyrtios* sp.	No applicable	South China Sea	No data	Wang et al., 2024 [246]
Hyrfarnediol A (**884**)	*Hyrtios* sp.	No applicable	South China Sea	Cytotoxicity against HCT-116	-
Ircinialactam J (**885**)	*Ircinia felix*	No applicable	Yucatan Peninsula, Mexico	Inhibition of HAdV5 infection	Ruiz-Molina et al., 2024 [247]
Ircinialactams K–L (**886–887**)	Ircinia felix	No applicable	Yucatan Peninsula, Mexico	No data	-

Table S4. Triterpenes and tetraterpenes (marked *) isolated from the marine sponges.

Compound	Sponge Species	Sponge-Derived Microbe	Sampling Location	Biological Activity	Ref.
Compound 888<br>Sipholenols J–L **(890–892)**<br>Siphonellinol D **(894)**<br>Siphonellinol C-23-hydroperoxide **(896)**	*Callyspongia siphonella*	No applicable	Red Sea	No data	Jain et al., 2009 [248]
Sipholenone E **(889)**<br>Sipholenol M **(893)**<br>Siphonellinol E **(895)**	*Callyspongia siphonella*	No applicable	Red Sea	P-gp modulatory activity	-

Rhabdastins A–C **(897–899)** Rhabdastoside A **(904)**	*Rhabdastrella globostellata*	No applicable	Amamio-shima Japan	No data	Hirashima et al., 2010 [249]
Rhabdastins D–G **(900–903)**	*Rhabdastrella globostellata*	No applicable	Amamio-shima Japan	Antiproliferative activity	-
Stelliferins J–N **(905–909)**	*Rhabdastrella cf. globostellata*	No applicable	Okinawa	No data	Tanaka et al., 2011 [250]
Globostelletins J–K **(910–911)**	*Rhabdastrella globostellata*	No applicable	South China Sea	Protein kinase inhibitors	Li et al., 2012 [251]
Globostelletins L–R **(912–918)**	*Rhabdastrella globostellata*	No applicable	South China Sea	No data	-
Stellettin N **(919)**	*Stelletta* sp.	No applicable	Hainan, China	No data	Xue et al., 2013 [252]
Jaspiferins C–F, H–J **(920–926)**	*Jaspis stellifera*	No applicable	South China Sea	No data	Xu et al., 2018 [253] Jin et al., 2014 [254]
Stellettins Q–R (**927–928**)	*Stelletta* sp.	No applicable	Vietnam	No data	Kolesnikova et al. 2021 [158]
Rhabdastins H–I (**929–930**)	*Rhabdastrella*	No applicable	Kenting, Taiwan	Active against K562 and Molt4	Lai et al., 2021 [255]
13-(*E*)-Geoditin A (**931**) 13-(*E*)-Isogeoditin B (**932**) 3-Acetylstelliferin D (**933**) 29-Acetylstelliferin D (**934**) Hainanstelletin A (**935**) Hainanstelletin B (**936**) 23,24-Ene-25-hydroxystelliferin D (**937**) 25,26-Ene-24-hydroxystelliferin D (**938**) Hainanstelletin C (**939**)	*zza globostellata*	No applicable	Ximao Island	Antibacterial activities	Chen et al., 2022 [256]
Erylosides R1, T1–T6 **(940–946)**	*Erylus formosus*	No applicable	Caribbean coast, Mexico	No data	Antonov et al., 2011 [257]
Sarasinosides N–R **(947–951)**	*Lipastrotethya* sp.	No applicable	Chuuk, Micronesia	Cytotoxicity against A549 and K562 cell lines	Lee et al., 2012 [258]
iso-D8646-2-6 **(952)**	*Callyspongia* sp.	Fungus *Epicoccum* sp. JJY40	Sanya, China	CPE and NF-κB inhibition	Peng et al., 2012 [259]
Urabosides F, A–B **(953–955)**	*Ectyoplasia ferox*	No applicable	Caribbean	No data	Colorado et al., 2013 [260]
Pouosides F, A–C, G, D, H, E, I **(956–964)**	*Lipastrotethya* sp.	No applicable	Micronesia	Cytotoxicity	Lee et al., 2011 [261]
Erylosides F_8, V_1, V_3, W_1–W_2 **(965, 966, 968, 970–971)**	*Erylus goffrilleri*	No applicable	Carribean	No data	Antonov et al., 2017 [262]
Eryloside V_2 **(967)** Eryloside W **(969)**	*Erylus goffrilleri*	No applicable	Carribean	Cytotoxicity and hemolytic activity	-

Melophluosides A–B (**972–973**)	*Melophlus sarassinorum*	No applicable	Siladen, Sulawesi	Cytotoxic against HeLa	Sadahiro et al., 2020 [263] Izzati et al., 2021 [264]
Gombaside A **(974)**	*Clathria gombawuiensis*	No applicable	Korea	Cytotoxicity and antibacterial	Woo et al., 2015 [224]
Coscinoderines A–J **(975–984)**	*Coscinoderma bakusi*	No applicable	Fannuk island, chuuk	No data	Tran et al., 2023 [260]
5,8-Epoxysarasinoside (**985**) 8,9-Epoxysarasinoside (**986**)	*Petrosia nigricans*	No applicable	Lipata, Philippines	Cytotoxic against HCT116 and A549	Mama et al., 2024 [266]
3-Oxo-7β,8β-epoxy-5α-lanost-24-en-30,9α-olide (**987**) 29-Nor-3-oxo-7β,8β-epoxy-5α-lanost-24-en-30,9α-olide (**988**) 3β-Acetoxy-7β,8β-epoxy-5α-lanost-24-en-30,9α-olide (**989**) 3β-Hydroxy-7β,8β-epoxy-5α-lanost-24-en-30,9α-olide (**990**)	*Penares* sp.	No applicable	Vietnam	No data	Kolesnikova et al., 2013 [267]
29-Nor-penasterone (**991**) 3β-Hydroxy-5α-lanosta-7,24-dien-30,9α-olide (**992**)	*Penares* sp.	No applicable	Vietnam	Cytotoxic against HL-60 cell	-
Stellettins W–X (**993–994**)	*Rhabdastrella globostellata*	No applicable	Cham Island	No data	Kozhushnaya et al., 2024 [268]
*Gukulenins A–B (**995–996**)	*Phorbas gukulensis*	No applicable	Korea	Cytotoxic	Park et al., 2010 [269]
*Compound **997**	*Luffariella variabilis*	No applicable	South China Sea	No data	Luo et al., 2021 [229]

282. Takeshi, Y.; Yoshihide, U.; Maiko, K.; et al. Determination of the Chemical Structures of Tandyukisins B–D, Isolated from a Marine Sponge-Derived Fungus. *Mar. Drugs*. **2015**, 13, 3231–3240. https://doi.org/10.3390/md13053231.
